# VTGAN based proactive VM consolidation in cloud data centers using value and trend approaches

**DOI:** 10.1038/s41598-025-04757-z

**Published:** 2025-06-20

**Authors:** Aya I. Maiyza, Hanan A. Hassan, Walaa M. Sheta, Karim Banawan, Noha O. Korany

**Affiliations:** 1 Informatics Research Institute, City of Scientific Research and Technological Applications (SRTA-City), Alexandria, Egypt; 2 Department of Electrical Engineering, Faculty of Engineering, Alexandria University, Alexandria, Egypt; 3 Electronics and Communications Engineering Department, American University in Cairo, Cairo, Egypt

**Keywords:** Cloud computing, Workload prediction, VM consolidation, GAN, LSTM, CloudSim, Electrical and electronic engineering, Computational science, Computer science, Information technology

## Abstract

Reducing energy consumption and optimizing resource usage are essential goals for researchers and cloud providers managing large cloud data centers. Recent advancements have demonstrated the effectiveness of virtual machine consolidation and live migrations as viable solutions. However, many existing strategies are based on immediate workload fluctuations to detect host overload or underload and trigger migration processes. This approach can lead to frequent and unnecessary VM migrations, resulting in energy inefficiency, performance degradation, and service-level agreement (SLA) breaches. Moreover, traditional time series and machine learning models often struggle to accurately predict the dynamic nature of cloud workloads. This paper presents a consolidation strategy based on predicting resource utilization to identify overloaded hosts using novel hybrid value trend generative adversarial network (VTGAN) models. These models not only predict future workloads but also forecast workload trends (i.e., the upward or downward direction of the workload). Trend classification can simplify the decision-making process in resource management approaches. We perform simulations using real PlanetLab workloads on Cloudsim to assess the effectiveness of the proposed VTGAN approaches, based on value and trend, compared to the baseline algorithms. The experimental findings demonstrate that the VTGAN (Up current and predicted trends) approach significantly reduces SLA violations and the number of VM migrations by 79% and 56%, respectively, compared to THR-MMT-PBFD. Additionally, incorporating VTGAN into the VM placement algorithm to disregard hosts predicted to become overloaded further improves performance. After excluding these predicted overloaded servers from the placement process, SLA violations and the number of VM migrations are reduced by 84% and 76%, respectively, compared to THR-MMT-PBFD.

## Introduction

In the realm of Cloud Computing, a paradigm shift has occurred, marked by the collaborative use of computing resources to cater to diverse services for end-users. Virtualization abstracts physical resources into virtual machines (VMs), streamlining management, reducing setup costs, and enabling flexible access^1^.

**Fig. 1:**
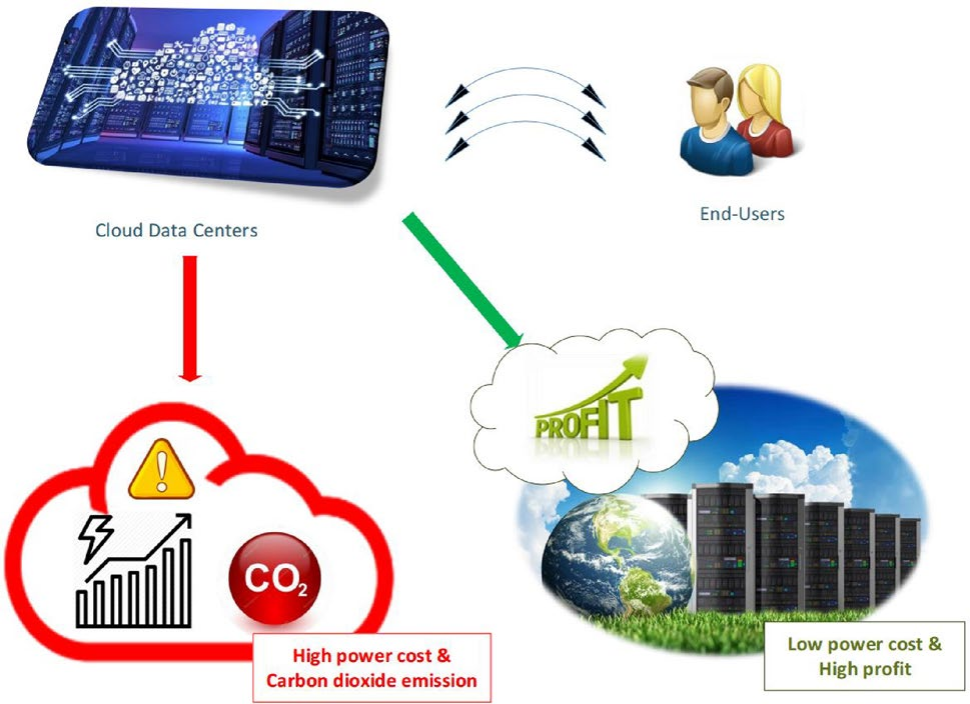
Green cloud data center.

As cloud adoption grows, optimizing resource utilization and reducing energy consumption (EC) in data centers have become essential^2,3^. Among data center subsystems, computing resources consume a significant portion of energy, making power management a major research focus in cloud computing, as shown in Fig. 1. Dynamic VM consolidation is a promising strategy to enhance energy efficiency by optimizing VM placement to minimize the number of active servers^4^. However, VM consolidation introduces challenges related to host performance and Quality of Service (QoS)^5,6^.

Resource management (RM) is crucial in cloud data centers. The key challenge is balancing energy efficiency and preventing service level agreement (SLA) violations. Frequent VM migrations and overloaded servers degrade QoS, while underutilized servers waste energy^7,8^. As a result, service providers require effective techniques for optimal RM^9,10^. Therefore, researchers focus on improving proactive techniques, predicting future workloads for VM migration decisions^11^. CPU utilization is a critical factor in power consumption, influencing energy efficiency^12^.

In prior work^13^, we introduced the VTGAN model and fine-tuned it to achieve optimal performance in predicting cloud workload values and trends. We then analyzed its predictive effectiveness relative to other machine learning (ML) and deep learning (DL) models. In this paper, we extend VTGAN to evaluate its feasibility in a real cloud environment by integrating it into a CloudSim-based overload detection algorithm for dynamic workload prediction. We comprehensively evaluate VTGAN under realistic cloud scenarios using PlanetLab workloads to measure its effectiveness in minimizing unnecessary VM migrations, maintaining energy efficiency, and reducing SLA violations. Furthermore, we incorporate VTGAN predictions into VM placement algorithms and demonstrate that excluding hosts predicted to become overloaded from placement decisions improves overall resource utilization and further enhances SLA compliance while reducing energy consumption.

The structure of this paper is as follows: “Background” section presents the related work. “Methods” section illustrates the proposed VTGAN approaches. “Results” section presents the experimental configurations of the evaluation conducted and the experimental findings. “Conclusion” section summarizes our conclusion of findings.

## Background

Research on RM and resource reallocation in cloud computing focuses on energy efficiency and workload optimization from multiple perspectives^10,14,15^. Early studies primarily addressed periodic workload redistribution without optimal timing algorithms^16,17^. Alternative methods introduced threshold-based heuristics for VM migrations^18,19^ and adaptive thresholds for overload detection^20^.

To enhance energy efficiency and resource utilization, several energy-aware RM techniques have been proposed. VM migration cost recovery strategies balance energy savings with migration overhead^21^, while aggregation-based VM placement improves resource efficiency with minimal performance degradation^22^. Sustainability models and computing frameworks further optimize scheduling and resource allocation^23^. Additionally, advanced VM placement strategies leveraging genetic algorithms enhance energy efficiency and workload distribution^24^. These studies contribute to the continuous improvement of energy-efficient RM in cloud environments.

Despite these advancements, many existing approaches remain reactive, responding to workload fluctuations rather than predicting future trends. This often leads to excessive VM migrations, increased energy consumption, and performance degradation. Furthermore, traditional time-series forecasting and ML models struggle to capture the dynamic nature of cloud workloads, resulting in suboptimal RM decisions.

Recent research has explored predictive models for VM consolidation and resource forecasting, utilizing linear regression, Markov models, and ML techniques^25–28^. While hybrid and ensemble-based approaches improve prediction accuracy, their real-world deployment remains limited^29,30^. Achieving accurate workload predictions in cloud environments remains a significant challenge. Table 1 summarizes various workload prediction methods applied in simulated cloud environments using CloudSim^31^, categorizing them based on learning technique, dataset, performance metrics, strengths, and weaknesses.

**Table 1:** Comparison of the workload prediction approaches for RM methods.

Authors	Technique used	Dataset	Performance metric	Strength	Weakness
Jheng et al.^32^	Grey		Power consumption (W)	Balances workload Lower power consumption	Unreliable predictions for fluctuating workloads
Hsieh et al.^33^	Grey–Markov	PlanetLab	EC (KWh) Number of VM migrations SLAV ESV	Prediction accuracy increases Effectively reduces number of migrations, EC, and SLAVs	Effective for short to medium term resource forecasting
Sayadnavard et al.^34^	e-MOABC	PlanetLab	EC (KWh) Number of VM migrations SLAV ESV	Reduces EC, resource wastage and enhances system reliability	Effective for short to medium term resource forecasting
Li et al.^27^	Naive Bayesian	PlanetLab	EC (KWh) Number of VM migrations Number of host shutdowns SLAV ESV PDM	Reduces SLA violation rates while maintaining energy efficiency	Require the prior probability that depends on some hypothesis
Fu and Zhou^35^	ARIMA	PlanetLab Google cluster	EC (KWh) Number of VM migrations SLAV	Reduce the EC, number of VM migrations and SLAVs	can not fit with long-term time-series data
Chehelgerdi-Samani and Safi-Esfahani^36^	ARIMA	PlanetLab	EC (KWh) Number of VM migrations SLAV Throughput (%)	Reduce the EC and number of VM migrations	Needs optimization to reduce SLAVs
Farahnakian et al.^25^	Linear regression	PlanetLab	EC (KWh)	Simple implementation Reduces EC and SLAVs	Effective for short-term resource forecasting and capturing linear relationships
Farahnakian et al.^37^	Linear regression	PlanetLab	EC (KWh) Number of VM migrations SLAV&ESV	Simple implementation Reduces EC and SLAVs	Effective for short-term resource forecasting and capturing linear relationships
Hieu et al.^38^	Linear regression	PlanetLab Google cluster	EC (KWh) Number of VM migrations SLAV	Simple implementation Reduces EC and network load	Effective for short-term resource forecasting and capturing linear relationships
Shaw et al.^39^	Linear regression	PlanetLab	EC (KWh) Number of VM migrations Number of overloaded nodes SLAV	Simple implementation Reduces EC and eliminates the repeated migration of the same VM	Effective for short-term resource forecasting and capturing linear relationships
Farahnakian et al.^40^	KNN	PlanetLab	EC (KWh) SLAV	Reduces EC and maintains required SLA	High complexity
Saxena and Singh^9^	OM-FNN	Google cluster	Power consumption (KW) Power saving (%)	Achieves the high data center energy efficiency and avoiding SLA violations	Require sufficient data Need optimization algorithm to train weights
Thein et al.^41^	RL-Fuzzy logic	PlanetLab	EC (kWh) Power usage effectiveness (PUE) SLAV	Reduces the total cost and increases the resource utilization	Training result is unstable
Ghobaei-Arani et al.^29^	Linear regression-RL	ClarkNet NASA	SLA Violation (%) Total cost ($) Profit ($)	Improves resource utilization and response time	Suitable only for predicting stationary future resource utilization
Liu et al.^42^	EQVC (ARIMA-based)	PlanetLab Bitbrains	-EC (KWh) Number of VM migrations SLAV ESV	Reduces EC, the number of VM migrations and QoS guarantees	Parameters must be carefully selected and defined
Rezakhani et al.^3^	ANN	PlanetLab	EC (KWh) Number of VM migrations SLAV ESV	Reduces EC, the number of VM migrations, and SLAVs	Need prediction accuracy study
Proposed work	VTGAN	PlanetLab	EC (KWh) Number of VM migrations SLAV ESV	Reduces unnecessary migration effectively	High training complexity

This work introduces a proactive consolidation strategy based on VTGAN^13^, a hybrid value-trend generative adversarial network model designed to predict both future workload values and trends. Unlike prior models that rely solely on historical data for overload detection, VTGAN integrates workload direction prediction, enabling more informed VM migration decisions and improving resource management efficiency.

## Methods

In this paper, we focus on Infrastructure-as-a-Service (IaaS), where cloud service providers dynamically allocate computing, storage, and network resources based on demand. The system consists of M heterogeneous hosts, each characterized by CPU capacity (measured in millions of instructions per second—MIPS), RAM, and bandwidth. Users are assigned VMs from the available hosts. To ensure flexibility across various scenarios, we do not impose assumptions on applications or workloads, considering CPU utilization as the primary workload determinant.

The VM consolidation process is divided into four key components: (1) detecting overloaded hosts, (2) selecting a VM for migration, (3) choosing a destination host, and (4) identifying underloaded hosts.

**Fig. 2:**
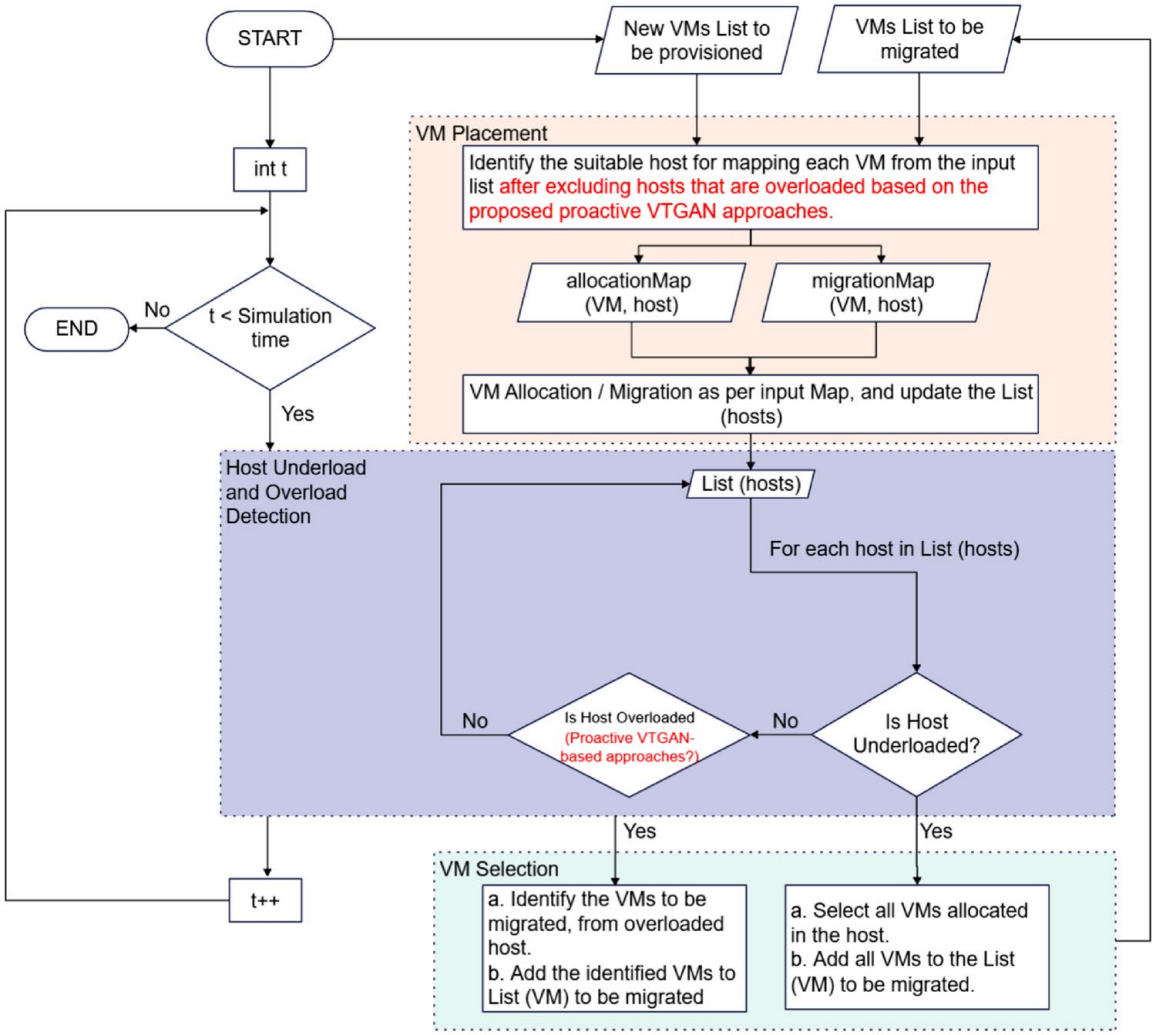
VTGAN-based diagram for VM consolidation, illustrating host overload detection and VM placement strategies for optimized resource utilization in cloud data centers. Our contributions are colored in red.

Figure 2 illustrates the VTGAN-based VM consolidation framework designed to optimize resource utilization and minimize unnecessary migrations in cloud data centers. The process begins with identifying VMs for provisioning and migration. VM placement follows the Power Best Fit Decreasing (PBFD) algorithm to ensure efficient allocation while proactively excluding hosts predicted to become overloaded. The overload detection mechanism leverages VTGAN-based forecasting to predict workload values and trends, thereby mitigating performance degradation and reducing excessive migrations. Upon detecting an overloaded host, VM selection adheres to the Minimum Migration Time (MMT) criterion to minimize downtime and enhance consolidation efficiency. Additionally, the underload detection mechanism identifies lightly loaded servers for consolidation, further reducing energy consumption. The red highlights in Fig. 2 indicate the specific contributions of this study in overload detection and VM placement, which are discussed in detail in this section.

The VTGAN model^13^ forecasts VM utilization to prevent host overload. Traditional analytical models struggle with hidden parameters, such as hardware design intricacies, making them impractical for next-generation processors with distinct architectures. In contrast, VTGAN effectively captures non-linear dependencies in cloud workloads, enhancing migration decision-making and offering a more adaptable, time-efficient solution when trained on suitable datasets.

**Fig. 3:**
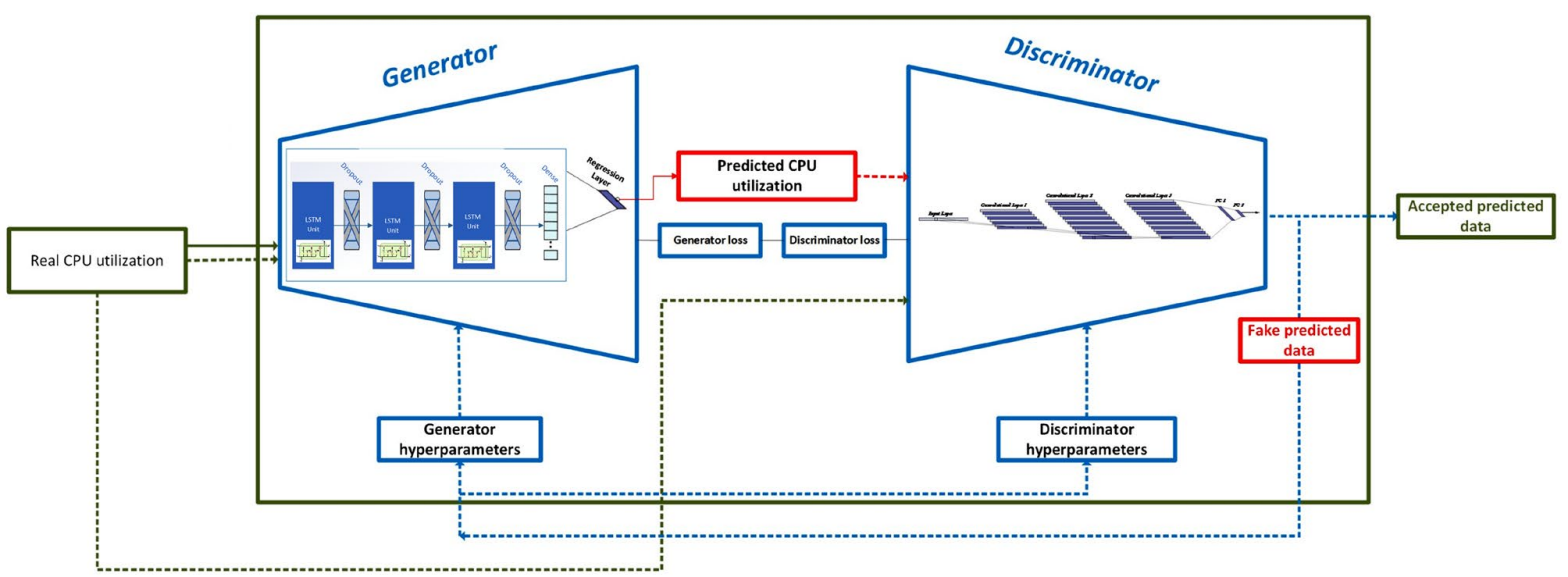
The proposed VTGAN model architecture. The generator, based on LSTM, captures temporal dependencies in CPU utilization traces, while the discriminator, implemented as a multilayer 1D-CNN, extracts essential temporal features for workload prediction and classification.

Figure 3 illustrates the proposed system utilizing the VTGAN model. This study uses LSTM as a generator to produce CPU traces. As highlighted in Ref.^13^, RNNs map generated data based on past input history, making them well-suited for sequential data processing. We specifically choose LSTM over GRU due to its superior performance on PlanetLab trace data for One-Step-Ahead prediction and classification. For the discriminator, we employ a multilayer 1D-CNN, chosen for its capability to extract temporal features, improving workload pattern recognition.

### Value approaches

In any dynamic VM consolidation process, it is essential to assess whether each host is overloaded. In these experiments, decisions are based exclusively on predicted near-future and/or current value, preventing unnecessary migrations and reducing the overall number of migrations.

Algorithm 1 implements an overload host detection policy that relies solely on predicted values. An optimal time window of three time units was utilized, as recommended by Ref.^13^. Therefore, the first three time slots were designated as a warm-up phase, following the structure outlined in Algorithm 1. During this phase, overloaded hosts were identified by comparing their current CPU utilization to a fixed threshold. The warm-up phase is particularly crucial when historical data is not available, such as at the beginning of the VM consolidation process. After this phase, a one-step-ahead VTGAN is used to predict the CPU utilization of every VM allocated to the tested host. A host is classified as overloaded if the sum of its predicted CPU utilization values meets or exceeds the established threshold.

**Algorithm 1:**
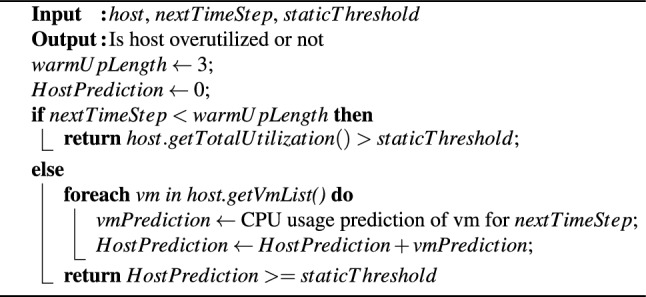
VTGAN-value overload host detection (predicted only).

Algorithm 2 implements a policy for detecting overloaded hosts by considering both predicted and current CPU utilization values. A host is classified as overloaded if the sum of its predicted and current CPU utilization values meets or exceeds a specified threshold. This approach prioritizes minimizing the number of VM migrations rather than solely focusing on reducing SLAV caused by overloaded servers. Additionally, we propose an alternative policy that identifies overloaded hosts based on either predicted or current CPU utilization values. This alternative is more suitable for service providers who need a system that is highly responsive to SLAV.

**Algorithm 2:**
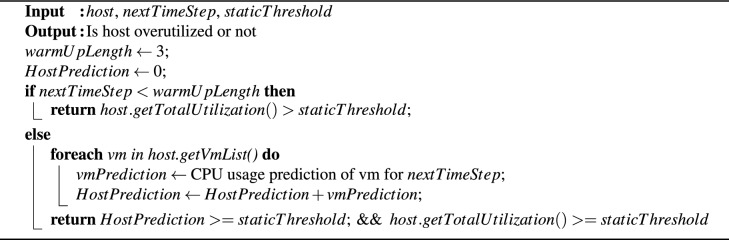
VTGAN-value overload host detection (predicted and current).

### Trend approaches

In these experiments, we base our decisions on the current CPU utilization value and the predicted trend. We categorize the expected direction of future CPU utilization changes as either upward or downward. An upward trend indicates that future CPU utilization is expected to exceed the current value, while a downward trend suggests a decrease. In many practical applications, understanding workload trends is more important than knowing exact values (e.g., in stock prediction). In this approach, a host is classified as overloaded only if its current CPU utilization meets or exceeds a predetermined threshold and the trend is upward, as illustrated in Algorithm 3. The upward trend is formally defined as follows:

Upward class:

1 $$\begin{aligned} C_h (t+1) - C_h (t) > 0, \end{aligned}$$

where $C_h (t+1)$ and $C_h (t)$ represent the predicted and current CPU utilization percentages, respectively. Algorithm 3 may more effectively reduce the number of migrations caused by overloaded hosts compared to Algorithm 2. That is because it takes into account scenarios where future CPU utilization is expected to exceed the threshold and follows an upward trend. In such cases, Algorithm 2 would initiate a VM migration, while Algorithm 3 provides the host with an opportunity to stabilize in subsequent iterations without requiring system intervention.

**Algorithm 3:**
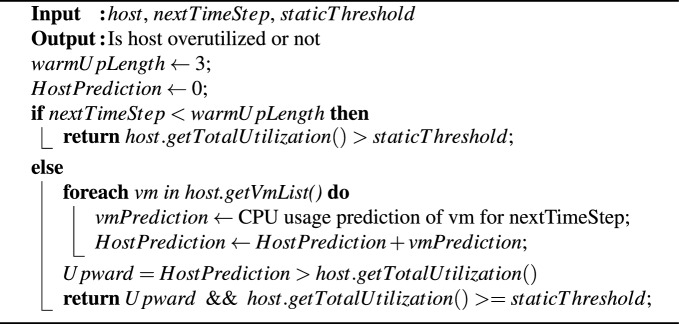
VTGAN-trend overload host detection.

To enhance control, we categorize the predicted CPU utilization trend using two classes: upward and non-upward. This approach is inspired by Financial time series modeling, as discussed in Ref.^43^. Consequently, a host is classified as overloaded only if its current CPU utilization meets or exceeds a specified threshold and the trend is classified as upward. This classification is regulated by a specific parameter ($\alpha$), as illustrated in Algorithm 4 and as follows:

Upward class:

2 $$\begin{aligned} C_h (t+1) - C_h (t) > \alpha , \end{aligned}$$

where $\alpha$ is a tuning parameter that controls the system. When $\alpha$ is set to zero, Algorithm 4 operates in the same manner as Algorithm 3. As $\alpha$ increases, the system perceives this as a movement toward the non-upward class. This adjustment is intended to reduce the number of migrations more effectively.

**Algorithm 4:**
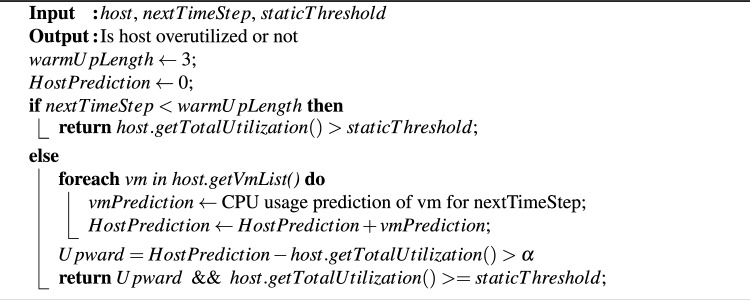
VTGAN overload host detection with $\alpha$ trend.

### VTGAN-trend approach with two adjacent trends

We conduct two experiments based on decisions derived from two adjacent trends, as follows:

- *VTGAN (up current and predicted trends):* A host is classified as overloaded only if its current CPU utilization meets or exceeds the threshold value of 80% and both the current and next predicted trends are upward.
- *VTGAN (two up predicted trends):* A host is classified as overloaded only if its current CPU utilization meets or exceeds the threshold value of 80%, and the two subsequent predicted trends are upward, as illustrated in Algorithm 5.

**Algorithm 5:**
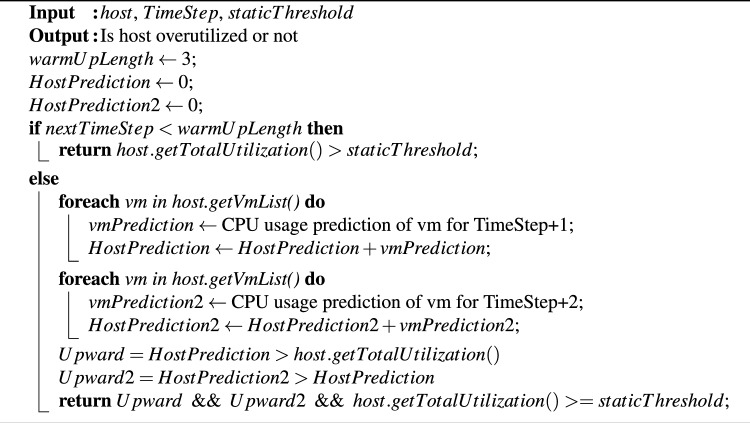
VTGAN-two predicted trend overload host detection.

### VTGAN-based approaches with different overload and placement algorithms

During the earlier approaches discussed in Sections “Value approaches”, “Trend approaches”, and “VTGAN-Trend approach with two adjacent trends”, we apply the VM placement algorithm to identify destination hosts for VMs selected for migration. The traditional Power-aware Best Fit Decreasing (PBFD) algorithm^20^ is used during the warm-up phase (with a length of 3), as no CPU value or trend prediction is available at that stage.

After the warm-up phase, we exclude hosts predicted to become overloaded based on the chosen overload detection algorithm, whether using VTGAN-Value or VTGAN-Trend approaches. For instance, if the overload detection algorithm follows the VTGAN approach (Predicted Overload), we first apply the PBFD algorithm. Then, we disregard hosts predicted to become overloaded based on the upcoming predicted value, as illustrated in Algorithm 6.

In these experiments, we examine the impact of modifying the VM placement algorithm to exclude hosts predicted to become overloaded based on different approaches. Therefore, we apply all six VTGAN-based overload detection algorithms and the THR baseline algorithm. We conduct seven experiments for each algorithm using the modified PBFD VM placement algorithm, disregarding hosts predicted to become overloaded based on the seven approaches (THR, three VTGAN-Value approaches, and three VTGAN-Trend approaches).

**Algorithm 6:**
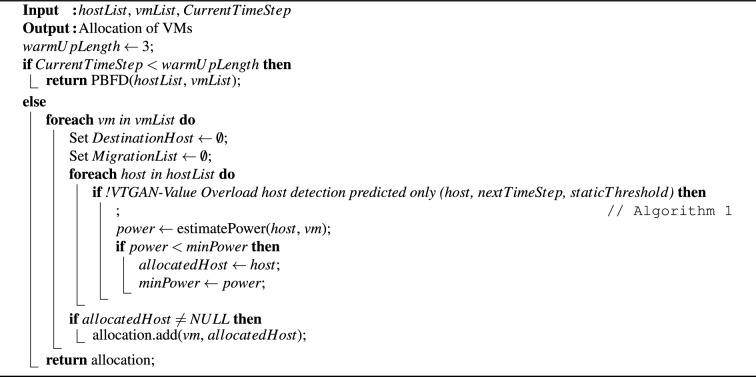
PBFD VM placement algorithm neglecting the proactive overloaded hosts.

## Results and discussion

This section presents the experimental setup and the results of the proposed VTGAN algorithms. Subsections “Results of VTGAN-Value approaches”, “Results of VTGAN-Trend approaches”, and “Results of VTGAN-Trend approach with two adjacent trends” illustrate the experimental results of the proposed approaches compared to baseline models using value, trend, and two adjacent trends approaches, respectively. Additionally, Section “Results of applying VTGAN approaches in PBFD VM placement algorithm” presents the effect of the modified placement algorithm based on different proactive VTGAN approaches.

### Experimental configuration and evaluation methodology

This subsection describes the experimental setup, the performance evaluation metrics, and the workload trace used in the study. These elements are essential for assessing the effectiveness of the proposed approaches. The evaluation involves comparing these approaches to traditional baseline methods.

For this purpose, the CloudSim toolkit^31^ has been chosen due to its capability to model cloud computing environments accurately. It is used to simulate the proposed approaches and compare their results against the traditional baseline methods, specifically THR-MMT-PBFD and LR-MMT-PBFD.

In our experiments, we simulate a heterogeneous data center featuring two different specifications. Table 2 details the configuration of the hosts. Additionally, Table 3 presents the characteristics of the VMs, while Table 4 illustrates the power consumption of the hosts at various utilization levels.

**Table 2:** The configuration of hosts^20^.

Hosts	CPU type	Freq.	Cores	RAM
		(MHz)		(GB)
HP ProLiant ML110 G4	Intel Xeon 3040	1860	2	4
HP ProLiant ML110 G5	Intel Xeon 3075	2660	2	4

**Table 3:** VM types^33^.

VM type	CPU (MIPS)	RAM (GB)
High CPU medium instance	2500	0.85
Large instance	2000	1.70
Small instance	1000	1.70
Micro instance	500	0.61

**Table 4:** Power consumption of hosts according to their CPU usage (in Watts)^33^.

Server	Sleep	0%	10%	20%	30%	40%	50%	60%	70%	80%	90%	100%
HP ProLiant G4	10	86	89.4	92.6	96	99.5	102	106	108	112	114	117
HP ProLiant G5	10	93.7	97	101	105	110	116	121	125	129	133	135

To ensure consistency in the experiments, we used a fixed set of traces for workload generation and iterative testing. The workload traces were sourced from the CoMon project, which is a monitoring framework for PlanetLab^44^. These traces include CPU utilization data recorded at 300-s intervals (or every 5 min) over a 24-h period from hundreds of VMs hosted on servers across more than 500 global locations. As presented in Table 5, data from ten different days between March 2011 and April 2011 were utilized. The VMs were operated by independent users running a variety of heterogeneous applications, which reflect a typical IaaS environment.

**Table 5:** Workloads data characteristics (CPU utilization)^33^.

Workloads	Date of workloads	Number of servers	Number of VMs	Mean (%)	St.dev. (%)	Quartile 1 (%)	Median (%)	Quartile3 (%)
W1	03/03/2011	800	1052	12.31	17.09	2	6	15
W2	06/03/2011	800	898	11.44	16.83	2	5	13
W3	09/03/2011	800	1061	10.70	15.57	2	4	13
W4	22/03/2011	800	1516	9.26	12.78	2	5	12
W5	25/03/2011	800	1078	10.56	14.14	2	6	14
W6	03/04/2011	800	1463	12.39	16.55	2	6	17
W7	09/04/2011	800	1358	11.12	15.09	2	6	15
W8	11/04/2011	800	1233	11.56	15.07	2	6	16
W9	12/04/2011	800	1054	11.54	15.15	2	6	16
W10	20/04/2011	800	1033	10.43	15.21	2	5	12

Our assumption is based on a linear relationship between the power consumption of a physical server and its CPU usage^45,46^. To ensure accurate power consumption data, we use real results obtained from the SPECpower benchmark^47^.

The experiments are performed on a system equipped with an Intel(R) Xeon(R) Gold 6248 processor running at a clock speed of 2.5 GHz, paired with 32 GB of memory. The DL models are implemented using the Keras framework with TensorFlow as the backend, utilizing CuDNN kernels for optimized performance. Table 6 outlines the architecture of the VTGAN model used in these experiments.

**Table 6:** The structure of VTGAN models^13^.

	Layers	Configuration
Generator parameters	Bidirectional cuDNNLSTM	256 units, dropout= 0.2
		L1 regularization for kernel, recurrent, and bias terms is ${10}^{-5}$
	cuDNNLSTM	128 units, dropout= 0.2
		L1 regularization for kernel, recurrent, and bias terms is ${10}^{-5}$
	cuDNNLSTM	128 units, dropout= 0.2
		L1 regularization for kernel, recurrent, and bias terms is ${10}^{-5}$
	FC	Dense, output units (1 for one-step-ahead)
		L1 kernel, and bias regularization = ${10}^{-5}$
Discriminator parameters	1D-CNN	flter=64, kernel size=5, strides=2, padding=same
		Leaky ReLU activation (alpha=${10}^{-3}$)
	1D-CNN	flter=128, kernel size=5, strides=2, padding=same
		Leaky ReLU activation (alpha=${10}^{-3}$)
	1D-CNN	flter=128, kernel size=5, strides=2, padding=same
		Leaky ReLU activation (alpha=${10}^{-3}$)
	Flatten	
	FC 1	Dense, units=64, LeakyReLU activation
	FC 2	Dense, output units (1 for one-step-ahead),
		Sigmoid activation

#### Performance evaluation metrics

The primary goals of the approaches proposed in this paper are to minimize unnecessary migrations, reduce EC, and limit SLAVs to maintain an acceptable level of QoS. The following eight metrics are utilized to evaluate their effectiveness.

1. *Total energy consumption (EC):* This metric represents the EC of physical hosts within the data center, which is directly influenced by the power usage of the CPU, memory, storage, and VM migrations^45,48^. Research has demonstrated that the power consumption of physical hosts can be modeled as a linear function of CPU utilization, as shown in Eq. (3)^49^.
  3 $$\begin{aligned} P(h)= K * P_{max} + (1 - K) \times P_{max} \times h^{u}. \end{aligned}$$
  $P_{max}$ denotes the maximum power required when the CPU of a specific host operates at full capacity (100% utilization). The constant *K*, identified as 0.7 through previous studies, represents the power consumption level of an idle host. Additionally, $h^u$ signifies the current CPU utilization of the host^50^. Given the variability in CPU utilization over time, the host’s CPU utilization can be expressed as a function of time. The total EC of a host is defined by Eq. (4).
  4 $$\begin{aligned} E= \int _{t_{0}}^{t_{1}} P(h^{u} (t)) {d}t. \end{aligned}$$
2. *Total number of VM migrations:* The number of VM migrations is influenced by the conditions of overloaded and underloaded servers. While live migration can help reduce downtime^51,52^, it negatively impacts VM performance, leading to an average degradation of about 10%^16^. The time required for migration and the related decrease in performance can be estimated using Eqs. (5) and (6)^20^.
  5 $$\begin{aligned} T_{m_{j}}= & \frac{M_{j}}{B_{j}}, \end{aligned}$$
  6 $$\begin{aligned} U_{d_{j}}= & 0.1 \times \int _{t_{0}}^{{t_{0}+T_j^m}} u_j (t) {d}t. \end{aligned}$$
  In Eq. (5), $M_j$ denotes the RAM usage of the VM, while $B_j$ represents the available network bandwidth. In Eq. (6), $U_{d_{j}}$ indicates the total performance degradation of VM *j*. The variables $t_0$ and $T^m_j$ correspond to the start and completion times of the migration, respectively, while $u_j$ denotes the CPU utilization of VM *j*. Since VM migration contributes to performance degradation, as shown in Eq. (6), reducing its frequency helps minimize SLA violations.
3. *SLA violation time per active host (SLATAH):* This metric represents the percentage of time active hosts operate at full CPU utilization (100%). Such conditions can severely degrade system performance, particularly when applications fully utilize the CPU’s capacity. As a result, VMs may fail to meet client demands^53^, leading to SLA violations. SLATAH is calculated using Eq. (7)^20^.
  7 $$\begin{aligned} SLATAH = \frac{1}{N} \sum _{i=1}^N \frac{T_{s_{i}}}{T_{a_{i}}}, \end{aligned}$$
  where *N* represents the number of hosts, $T_{s_i}$ denotes the total time duration that host *i* operated at 100% utilization, resulting in QoS degradation, while $T_{a_{i}}$ represents the total time that host *i* remained active.
4. *Performance degradation due to VM migration (PDM):* The overall performance degradation of VMs due to migrations can be calculated using Eq. (8):
  8 $$\begin{aligned} PDM = \frac{1}{M} \sum _{j=1}^M \frac{C_{d_j}}{C_{r_j}}, \end{aligned}$$
  where *M* represents the number of VMs, $C_{d_j}$ denotes the estimated performance degradation of VM *j* caused by migrations, and $C_{r_j}$ represents the total CPU capacity required by VM *j* during its active period. This assessment measures the VM’s performance before and during the migration process. Similar to the previously mentioned metric, it negatively affects the system’s ability to meet application performance requirements, ultimately leading to SLAV.
5. *SLA Violation (SLAV):* SLAs define performance metrics such as minimum CPU capacity and maximum response time, varying across applications. This study defines SLA compliance as consistently meeting 100% of performance requirements. SLA violations in IaaS are measured using SLATAH and PDM. The SLAV metric integrates both, capturing performance degradation from overload and migrations^54^. This relationship is represented in Eq. (9).
  9 $$\begin{aligned} SLAV = SLATAH \times PDM. \end{aligned}$$
6. *Energy and SLA violations (ESV):* This metric captures both EC and SLAV, which the resource management (RM) system aims to minimize. It is computed as shown in Eq. (10)^20^.
  10 $$\begin{aligned} ESV = EC \times SLAV. \end{aligned}$$
7. *Energy-SLA-migration (ESM):* This metric evaluates the simultaneous minimization of EC, SLA violations, and VM migrations. It is computed by multiplying EC with the product of SLAV and the total number of migrations, as illustrated in Eq. (11)^55^.
  11 $$\begin{aligned} ESM = E \times SLAV \times Number\ of\ VM\ migrations. \end{aligned}$$
8. *The number of host shutdowns:* This metric measures the number of underloaded hosts that transitioned into sleep mode after VM migration^56^.

### Results of VTGAN-value approaches

To assess the proposed VTGAN-Value approaches, as described in detail in Section “Value approaches”, we compare the results with the baselines from related overload detection algorithms, namely THR and LR. Table 7 presents the results obtained for the metrics described in Section “Performance evaluation metrics”.

**Table 7:** Performance evaluation using PlanetLab with seven metrics using VTGAN-value approaches.

Approach	EC	Number of migrations	SLATAH	PDM	SLAV	ESV	ESM
	(KWh)		(%)	(%)	$\times (10^{-5})$	$\times (10^{-3})$	$\times (10^2)$
THR	193.526	27,247.7	5.056	0.071	3.597	6.905	1.921
LR	166.46	29,142.5	6.218	0.083	5.185	8.543	2.541
VTGAN (predicted overload)	172.468	22,421.5	5.04	0.049	2.4727	4.2	0.954
VTGAN (predicted and current overload)	**164.727**	**18914.6**	5.007	**0.038**	**1.927**	**3.145**	**0.613**
VTGAN (predicted or current overload)	189.065	25108.7	**4.781**	0.064	3.071	5.739	1.467

These values represent the averages obtained from executing each algorithm using PlanetLab workloads. The results show that the proposed approaches effectively balance migrations and SLA compliance by leveraging VTGAN-Value methodologies to predict future host workloads. As a result, they outperform baseline methods across most metrics, though EC reduction remains minimal.

The overload detection algorithm considers a host overloaded only if both the predicted and current values indicate an overloaded state. This approach outperforms others (bold results) by minimizing unnecessary migrations and reducing PDM. Allowing cloud workloads to stabilize without immediate RM system intervention helps prevent performance degradation caused by migration and network overhead. However, if intervention is needed in subsequent iterations, migrations are performed to resolve SLA violations.

**Fig. 4:**
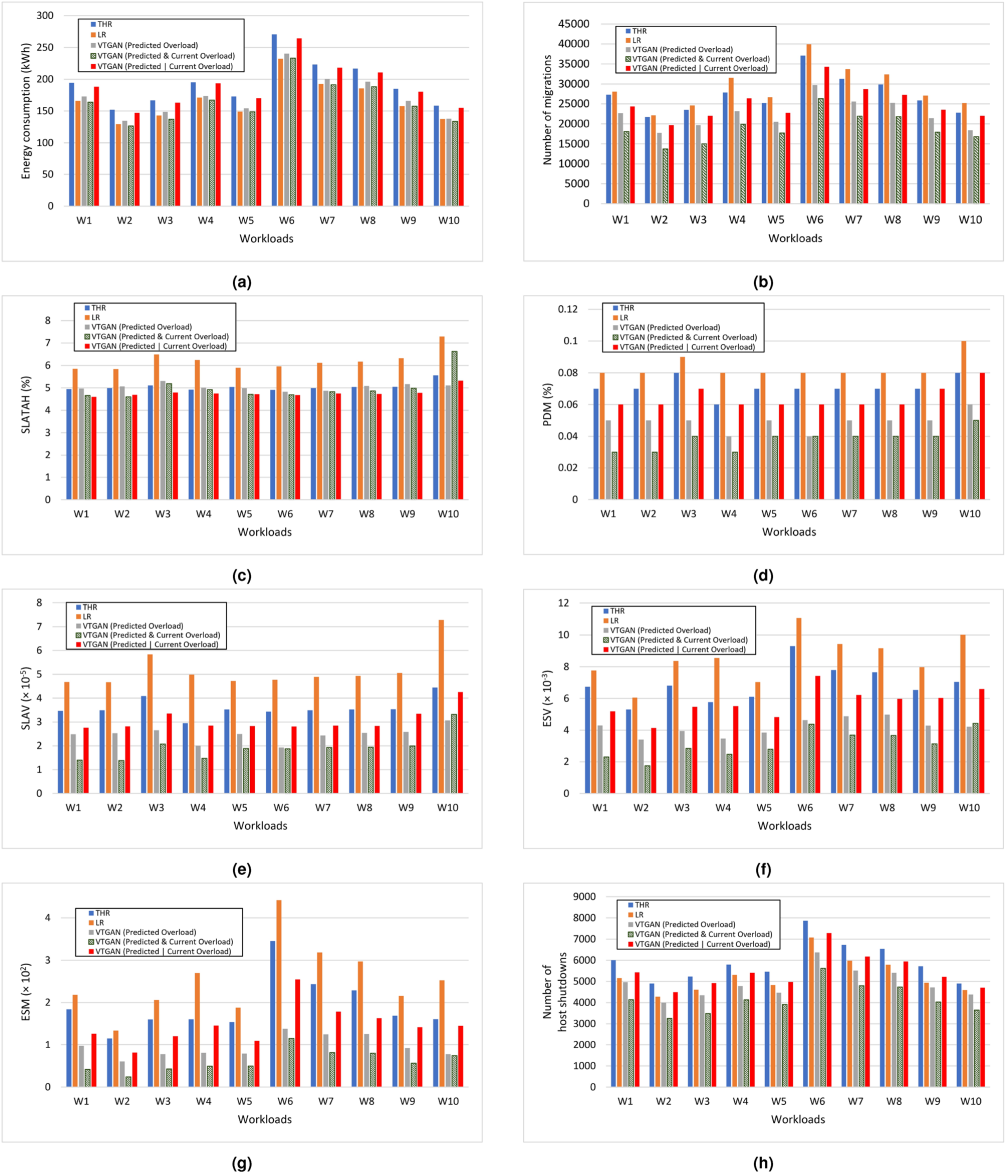
Performance comparison of the proposed VTGAN-Value approaches against baseline methods across eight evaluation metrics over ten workloads. Each workload is analyzed over a 24-h window. The subfigures illustrate (**a**) energy consumption, (**b**) number of VM migrations, (**c**) SLA violation time per active host (SLATAH), (**d**) performance degradation due to migrations (PDM), (**e**) SLA violations (SLAV), (**f**) energy-SLA violation (ESV), (**g**) energy-SLA-Migration (ESM), and (**h**) number of host shutdowns.

To assess VTGAN-Value approaches across different workloads, Fig. 4 presents a comparison of results over 10 days, with each workload evaluated over a 24-h period. These findings align with the average values shown in Table 7.

The VTGAN approach (predicted and current overload) demonstrates the lowest number of migrations and PDM compared to other approaches (Fig. 4b,d). Across almost all workloads (except W10), these results cause a reduction in SLAV, ESV, and ESM (Fig. 4e–g). This signifies that our VTGAN approach (predicted and current overload) effectively balances power cost and QoS guarantee. In W10, the VTGAN approach (predicted overload) records minimal values of SLAV and ESV due to prolonged periods of host overload compared to other workloads (Fig. 4c). However, considering the number of migrations, ESM records the lowest value across all workloads using the VTGAN approach (predicted and current overload), as illustrated in Fig. 4g.

Figure 4h shows that the VTGAN approach (predicted and current overload) considerably reduces the number of host shutdowns for all workloads. This decrease may be due to the effectiveness of this approach in minimizing the probability of migrations from overloaded servers that cause the activation of sleep hosts. As a result, in subsequent iterations, these hosts are identified as underloaded servers, prompting the system to migrate VMs to hosts in a normal state.

### VTGAN-trend approaches results

This section evaluates the performance of Algorithm 3 with the VTGAN-Trend overload detection technique. Additionally, we analyze the impact of adjusting the $\alpha$ parameter to filter out minor upward trends using Algorithm 4, as detailed in Section “Trend approaches”.

**Fig. 5:**
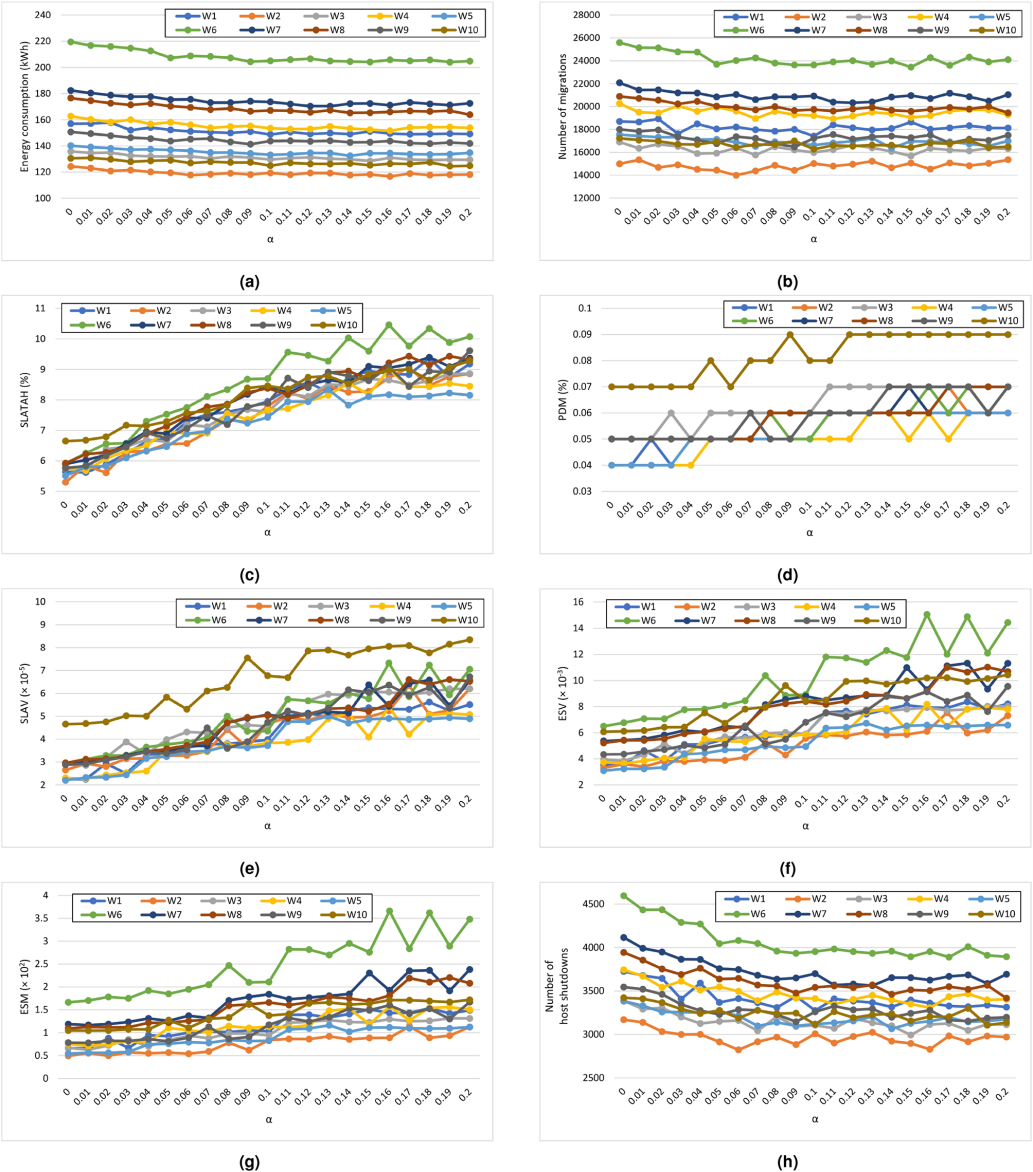
Performance comparison of VTGAN approach (Up Trend) across eight key performance metrics for different values of $\alpha$ (ranging from 0 to 0.2) for the ten workloads. The subfigures represent (**a**) Energy consumption, (**b**) Number of VM migrations, (**c**) SLA violation time per active host (SLATAH), (**d**) Performance degradation due to migrations (PDM), (**e**) SLA violations (SLAV), (**f**) Energy-SLA violation (ESV), (**g**) Energy-SLA-Migration (ESM), and (**h**) Number of host shutdowns. These comparisons highlight the impact of different $\alpha$ values on system performance.

Figure 5 compares the results for the ten workloads, each assessed with $\alpha$ values ranging from 0 (Algorithm 3) to 0.2.

With small $\alpha$ values, there is only a slight improvement in energy savings and the number of migrations (Fig. 5a,b) as $\alpha$ increases. However, SLATAH significantly increases (Fig. 5c), leading to no overall improvement in SLAV, ESV, or ESM (Fig. 5e–g). Additionally, host shutdowns decrease notably (Fig. 5h) due to more migrations caused by underloaded hosts.

For large $\alpha$ values, all QoS indicators decline significantly (Fig. 5c–g) without improving energy savings, the number of migrations, or the number of host shutdowns (Fig. 5a,b,h).

This experiment (Algorithm 4) aims to refine trend classification by predicting CPU utilization trends of a host rather than for individual VMs, using three classes: (i) upward trend, (ii) hold, and (iii) downward trend. However, results indicate that this approach remains ineffective even for small $\alpha$ values.

### VTGAN-trend approach with two adjacent trends results

This section evaluates the performance of the VTGAN-Trend overload detection technique using two adjacent trends, as detailed in Section “VTGAN-Trend approach with two adjacent trends”.

Table 8 presents the average metric results (described in Section “Performance evaluation metrics”) using PlanetLab workloads, comparing the two-adjacent-trends approach with baseline methods and VTGAN-Value approaches.

**Table 8:** Performance evaluation using PlanetLab with seven metrics using VTGAN-trend approaches.

Approach	EC	Number of migrations	SLATAH	PDM	SLAV	ESV	ESM
	(KWh)		(%)	(%)	$\times (10^{-5})$	$\times (10^{-3})$	$\times (10^2)$
THR	193.526	27,247.7	5.056	0.071	3.597	6.905	1.921
LR	166.46	29,142.5	6.218	0.083	5.185	8.543	2.541
VTGAN (predicted overload)	172.468	22,421.5	5.04	0.049	2.4727	4.2	0.954
VTGAN (predicted and current overload)	164.727	18,914.6	5.007	0.038	1.927	3.145	0.613
VTGAN (predicted or current overload)	189.065	25,108.7	4.781	0.064	3.071	5.739	1.467
VTGAN (up trend)	157.991	19,224	5.816	0.049	2.872	4.514	0.89
VTGAN (up current and predicted trends)	160.655	**11917.4**	**2.92**	**0.026**	**0.768**	**1.231**	**0.153**
VTGAN (Two up predicted trends)	**149.159**	13,189.4	4.891	0.034	1.6748	2.474	0.333
VTGAN (predicted, current overload,	142.25	11,452.2	11.774	0.039	4.591	6.512	0.769
Up future trend, and up current trend)							

Before analyzing the results of two adjacent trends, Section “Results of VTGAN-Trend approaches” presents the results of VTGAN (Up Trend), which is based on a single predicted trend with $\alpha$ set to 0 (Algorithm 3). While VTGAN (Up Trend) outperforms THR and LR methods in most metrics (except SLATAH), VTGAN (Predicted and Current Overload) remains superior across all evaluated metrics except energy savings.

Overall, incorporating two consecutive trends in the VTGAN approach leads to significant improvements in energy savings, network overhead, and SLA compared to baseline and VTGAN-Value approaches, as shown in Table 8.

Experimental results confirm that the VTGAN approach (Up Current and Predicted Trend) outperforms all other methods in most performance metrics, demonstrating its overall efficiency.

Table 8 shows that the proposed VTGAN approach (Up Current and Predicted Trend) achieves the lowest number of VM migrations, averaging 11,917.4. In comparison, the baseline approaches record significantly higher averages of 27,247.7 and 29,142.5, demonstrating the superiority of the proposed method in reducing unnecessary migrations. This reduction also results in a slight decrease in EC by 17% and 3% compared to the baseline algorithms. Additionally, this proposed approach significantly reduces SLATAH by 42% and 53% compared to the baseline methods.

Reducing VM migrations optimizes CPU utilization, lowering PDM. VTGAN’s trend prediction minimizes overloaded hosts and unnecessary migrations, achieving a 63% and 69% PDM reduction compared to baseline algorithms.

The proposed method enhances resource availability by preventing host overload, reducing SLA violations by 79% and 85% compared to baseline algorithms, respectively, as shown in Table 8. Identifying overloaded hosts intelligently prevents frequent shutdowns (2834.6), reducing unnecessary migrations and improving efficiency while mitigating SLA violations and conserving energy.

The ESV metric offers valuable insights into EC and SLAV parameters. As shown in Table 8, the proposed approach consistently outperforms others, ensuring high QoS and energy savings. Additionally, it reduces operational costs, benefiting both end users and service providers, with ESV improving by 82% and 86% compared to the baseline algorithms, respectively. The ESM metric, which incorporates VM migrations into ESV, provides a comprehensive performance assessment. It improved significantly by 92% and 94% compared to the baseline algorithms.

Although the VTGAN approach (2 Up Predicted Trends) achieves the lowest EC value, we do not recommend its use. Our findings show that one-step-ahead prediction, whether for value or trend approaches, is more accurate than multi-step-ahead predictions^13^.

Finally, we combined the two best approaches, VTGAN (Predicted and Current Overload) and VTGAN (Up Current and Predicted Trends), to minimize the number of migrations. This method allows migrations only from overloaded servers when both current and predicted values exceed 80%, and trends are increasing. As shown in the last record of Table 8, the combined approach slightly reduces EC and the number of migrations compared to VTGAN (Up Current and Predicted Trends). However, this comes at the expense of performance degradation, with SLATAH increasing to 11.774%, compared to 2.92% for VTGAN (Predicted and Current Overload) and 5.007% for VTGAN (Up Current and Predicted Trends). Therefore, this approach will not be considered in future work.

The experimental results show that integrating VTGAN into both overload detection and VM placement improves performance compared to other proposed and baseline algorithms. VTGAN enables forecasting CPU utilization trends, allowing the system to detect potential overloads based on both predicted and current values. In VM placement, it helps identify hosts likely to become overloaded, ensuring VMs are migrated only to stable hosts. This approach optimizes resource utilization, reducing data center costs and enhancing QoS for end users while paving the way for advancements in cloud services.

### Results of applying VTGAN approaches in PBFD VM placement algorithm

This section examines the impact of the modified PBFD placement algorithm using the seven proposed VTGAN approaches, as detailed in Section “VTGAN-based approaches with different overload and placement algorithms”.

**Fig. 6:**
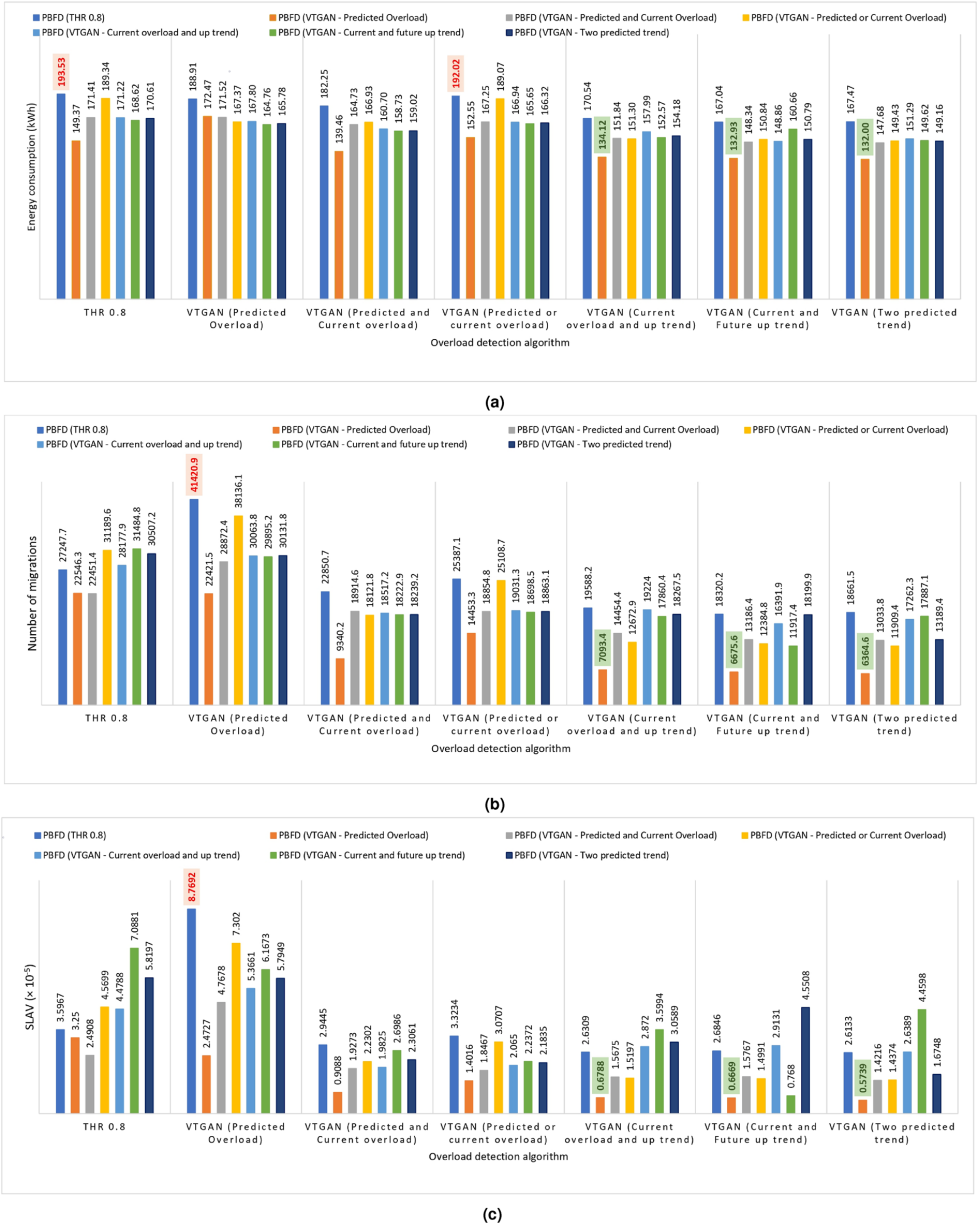
Performance comparison of different overload detection and placement algorithms combinations. The X-axis represents the overload detection approach, while the color variations indicate the modified PBFD placement algorithms. The subfigures illustrate (**a**) Energy consumption (EC), (**b**) Number of VM migrations, and (**c**) SLA violations (SLAV).

**Fig. 7:**
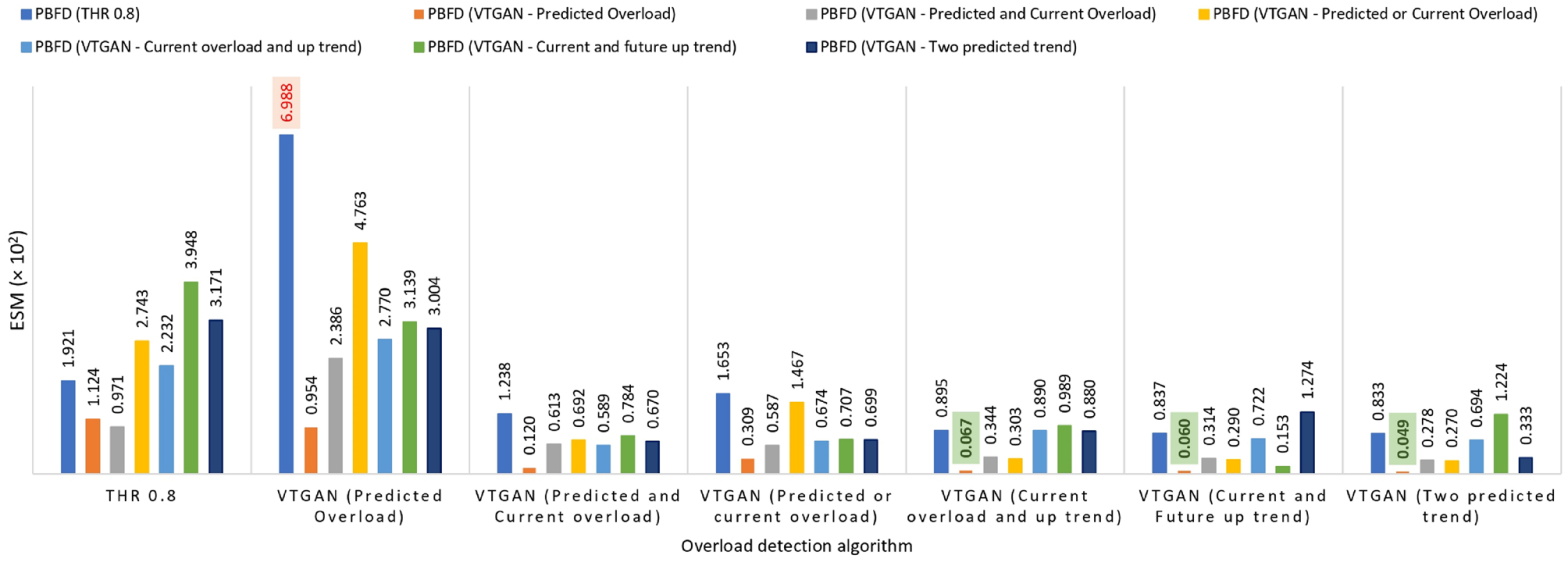
ESM for different combinations of overload detection and placement algorithms. The X-axis represents the overload detection approach, while the color variations indicate the modified PBFD placement algorithms.

Fig. 6 compares different overload and placement algorithm combinations. This figure highlights EC, the number of migrations, and SLAV as key performance metrics. Additionally, Fig. 7 presents ESM results for the same combinations, offering a comprehensive performance overview. These results represent average values from ten-day workload experiments.

Using the PBFD algorithm in its default state, combined with the THR algorithm for VM placement based on the current workload, generally results in the highest EC among all VTGAN approaches, as illustrated in Fig. 6a (blue bars). However, this method also leads to an increased number of migrations (Fig. 6b), as the placement algorithm selects destination hosts with the highest available CPU utilization. This can often result in those hosts becoming overloaded after migration, triggering further migrations.

The worst combination occurs when using VTGAN (Predicted Overload) for overload detection and PBFD (THR) for placement, leading to the highest number of migrations (Fig. 6b) and the worst SLAV (Fig. 6c) and ESM (Fig. 7) values. This outcome is expected, as a proactive overload detection algorithm conflicts with a reactive placement strategy.

In contrast, using the proactive VTGAN (Predicted Overload) PBFD placement algorithm enhances system performance by reducing unnecessary migrations and SLAV, particularly for VTGAN-Trend overload detection, while maintaining a balanced EC (Figs. 6, 7). The best combinations involve one of the three proposed VTGAN-Trend approaches paired with VTGAN (Predicted Overload) PBFD placement. These combinations significantly reduce SLA violations and the number of migrations by 84% and 76%, respectively, compared to THR-MMT-PBFD, while sustaining energy efficiency.

These results can be attributed to the VTGAN (Predicted Overload) PBFD placement algorithm, which selects hosts predicted to remain stable after migration. In contrast, the VTGAN (Predicted and Current Overload) PBFD algorithm imposes stricter constraints, reducing migrations by limiting available hosts. Meanwhile, the VTGAN (Predicted or Current Overload) PBFD algorithm may offer fewer destination hosts, causing more frequent reactivation of sleep-mode servers and leading to more migrations from underloaded hosts.

Table 9 presents a comparative analysis of our proposed VTGAN approach (Two UP Predicted Trends) and PBFD (VTGAN—Predicted Overload) against state-of-the-art methods with the same CloudSim configuration, as listed in Tables 2, 3, 4, and 5. The energy-efficient and QoS dynamic virtual machine consolidation (EQVC)^42^ method, based on the ARIMA model, achieves the lowest energy consumption (EC) of 111.52 KWh and the lowest energy SLA violation (ESV) of $0.67 \times 10^{-3}$, while the ADT-EC^57^ method, which uses adaptive dynamic thresholds based on EC levels, achieves the fewest migrations (4261.6). In contrast, VTGAN provides a balanced trade-off across these metrics, achieving the lowest SLAV at $0.5739 \times 10^{-5}$. Although VTGAN does not achieve the absolute lowest EC or migration count, it maintains a competitive EC value (131.996 KWh) and a reasonable migration rate (6364.6), optimizing both energy efficiency and system stability. These results highlight VTGAN’s effectiveness in minimizing SLAV by preventing prolonged host overloading while maintaining acceptable EC and migration rates, making it a strong alternative to existing approaches.

**Table 9:** Comparison between the best proposed VTGAN approaches and state-of-the-art.

Approach	EC	Number of migrations	SLAV	ESV
	(KWh)		$\times (10^{-5})$	$\times (10^{-3})$
EQVC^42^	**111.52**	6552	0.638	**0.67**
ADT-EC^57^	133.3	**4261.6**	0.75	0.99
Proposed model—VTGAN (Two UP Predicted Trends) &	131.996	6364.6	**0.5739**	0.71
PBFD (VTGAN—Predicted Overload)				

Significant values are in bold.

### Complexity analysis

To compute the time complexity of the VTGAN model described in Table 6, we leverage the analysis provided by Behera et al.^58^. The complexity analysis considers the total parameters for different neural network architectures. For the LSTM architecture, with 3 gates and a cell state, the total parameters can be expressed as: $C_{LSTM} = 4TQ + 4Q^2 + 3Q + QW$^59^, where *T* is the number of inputs, *Q* is the number of units in the hidden layer, and *W* is the number of outputs. The generator consists of a Bidirectional cuDNNLSTM and two cuDNNLSTM layers with the following configurations: (1) Bidirectional cuDNNLSTM: 256 units, (2) cuDNNLSTM: 128 units, (3) cuDNNLSTM: 128 units, and (4) Fully Connected (FC) Layer: Dense, output units (1 for one-step-ahead prediction). The complexity for a single BiLSTM layer is doubled: $C_{BiLSTM} = 2 \times C_{LSTM} = 2 \times (4TQ + 4Q^2 + 3Q + QW)$. For the first BiLSTM layer with 256 units: $C_{BiLSTM1} = 2 \times (4T \times 256 + 4 \times 256^2 + 3 \times 256 + 256W)$. For the first and second LSTM layers with 128 units: $C_{LSTM2} = C_{LSTM3} = 4T \times 128 + 4 \times 128^2 + 3 \times 128 + 128W$. The fully connected layer complexity is $C_{FC} = QW$. Combining the generator’s complexity: $C_{Generator} = C_{BiLSTM1} + C_{LSTM2} + C_{LSTM3} + C_{FC}$. The discriminator consists of 1D-CNN layers and fully connected layers: (1) 1D-CNN with 64 filters, (2) 1D-CNN with 128 filters (twice), (3) Flatten layer, (4) Fully connected layer (64 units, Leaky ReLU activation), and (5) Fully connected output layer (1 unit, sigmoid activation). The complexity for each 1D-CNN layer is $C_{CNN} = F \times K \times S$, where *F* is the number of filters, *K* is the kernel size, and *S* is the stride. Combining all the discriminator layers: $C_{Discriminator} = C_{CNN1} + C_{CNN2} + C_{CNN3} + C_{FC1} + C_{FC2}$. The final complexity of VTGAN is $C_{VTGAN} = C_{Generator} + C_{Discriminator}$. Considering $\Theta$ epochs of training: $C_{Model} \approx O(Q(Q + W) \times \Theta )$. Therefore, the complexity during the operational mode with 3 workload inputs and 1 predicted output can be calculated as $C_{Model} \approx O(3(3 + 1) \times 1) = O(12)$.

In order to describe the time complexity of the VTGAN-Value overload detection algorithm (Algorithm 1), we analyze its sub-components. Each algorithm involves CPU usage prediction for each virtual machine (VM) and aggregating these predictions to assess the host’s utilization. Assuming there are *N* hosts and *M* VMs, the time complexity for evaluating each host is *O*(*M*) as the algorithm iterates through each VM to predict CPU utilization. The final step, comparing the aggregated CPU usage prediction with the static threshold, is a constant-time operation, *O*(1). Therefore, the total time complexity for a single host evaluation is *O*(*M*), and for *N* hosts, it becomes *O*(*NM*).

For other algorithms, the additional checks for current utilization, upward trend detection, and trend difference with a threshold are constant-time operations, *O*(1), which do not affect the overall complexity. Thus, the complexity remains *O*(*M*) for a single host and *O*(*NM*) for *N* hosts.

In the case of the PBFD VM Placement Algorithm, the complexity analysis involves checking each host’s predicted overload status and estimating the power consumption for each VM placement. The overall complexity is *O*(*NM*).

### Feasibility of practical implementation in real cloud data centers

The practical deployment of VTGAN-based algorithms in real cloud data centers requires further evaluation, considering factors such as computational overhead, integration with cloud orchestration frameworks, and scalability. While deep learning models like VTGAN demand significant resources during training, inference can be optimized using hardware accelerators (e.g., GPUs, TPUs) to enable real-time decision-making with minimal latency.

Integrating VTGAN into open-source cloud management frameworks such as OpenStack is a critical step toward real-world adoption. OpenStack provides robust resource allocation and scheduling capabilities, making it a suitable platform for testing VTGAN-based consolidation strategies. Additionally, OpenStack Neat^60^, an extension designed for dynamic VM consolidation, serves as a foundation for incorporating VTGAN’s predictive workload modeling to enhance VM placement and host overload detection.

**Fig. 8:**
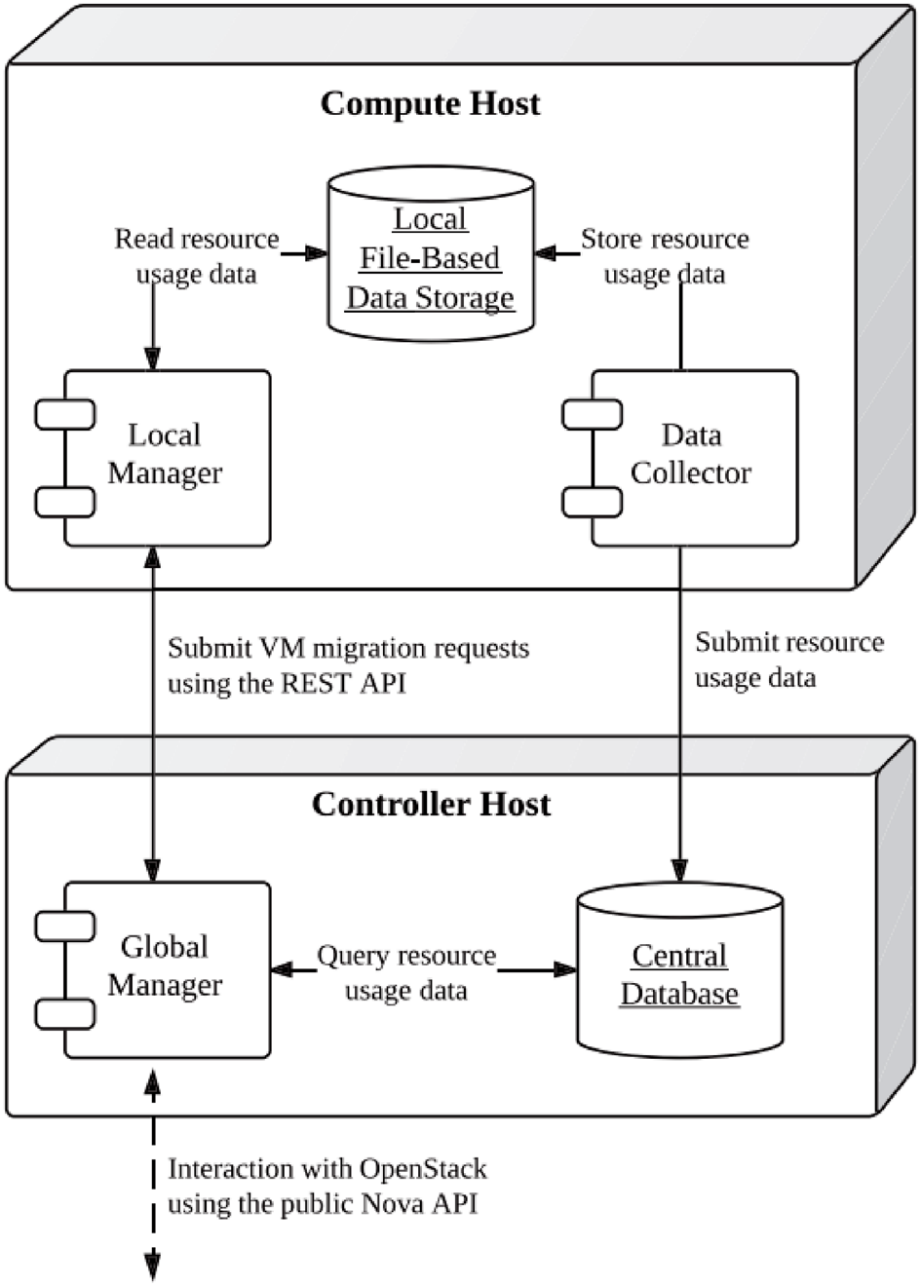
The deployment diagram of OpenStack Neat^60^.

VTGAN’s overload detection mechanism can be integrated into the local cloud infrastructure manager to optimize resource management further. As shown in Fig. 8, the local manager monitors host resource utilization and facilitates dynamic consolidation decisions^60^. Embedding VTGAN’s predictive modeling enables proactive detection of potential overloads, minimizing unnecessary VM migrations and improving energy efficiency. Unlike traditional threshold-based heuristics, which often lead to frequent migrations, VTGAN leverages workload forecasting to enhance decision-making and system adaptability.

Recent studies have demonstrated the feasibility of integrating predictive consolidation algorithms into OpenStack Neat^10,61,62^, highlighting the potential for incorporating deep learning-based solutions in cloud orchestration frameworks. Future work should focus on optimizing communication between local managers and global orchestrators to ensure seamless VM consolidation and real-time responsiveness in large-scale cloud environments.

## Conclusions

The dynamic nature of cloud workloads has emphasized the significance of efficient resource management and energy conservation. Various methods have been proposed to address these challenges, including VM live migration from overloaded hosts. This research introduces a novel approach based on VTGAN algorithms, a modified version of GAN, to predict workload value and trend^13^. LSTM is used as a generator to analyze past CPU utilization, while multiple layers of 1D-CNN function as the discriminator, extracting temporal features from sequential data.

Using VTGAN, we assess different approaches based on value and trend using actual workload data from PlanetLab, comparing them to baseline algorithms such as THR and LR. Results indicate that the VTGAN approach (Up current and predicted trend) significantly reduces unnecessary migrations, enhances QoS, and mitigates SLA violations while maintaining energy efficiency. By integrating VTGAN into overload detection and VM placement, the system can forecast CPU utilization trends and prevent selecting hosts that are likely to become overloaded. Experimental results show that the VTGAN approach (Up current and predicted trends) decreases SLA violations and VM migrations by 79% and 56%, respectively, compared to THR-MMT-PBFD. Furthermore, excluding overloaded servers from placement reduces SLA violations and VM migrations by 84% and 76%, respectively.

Through comprehensive experimentation, we have validated the scalability of VTGAN, demonstrating its capacity to handle progressively larger workloads with minimal computational overhead. By leveraging predictive modeling, the system proactively allocates resources, thereby reducing unnecessary virtual machine migrations and preventing host overload. Moreover, the model is designed to accommodate heterogeneous VM types and server configurations, ensuring broad applicability across diverse cloud environments. While our findings confirm scalability with increasing numbers of hosts and VMs. Future investigations will extend these evaluations to larger infrastructures and refine scheduling policies for heterogeneous data centers to further improve resource management efficiency. In addition, we plan to explore the trade-offs between computational complexity and prediction accuracy to optimize overall system performance.

VTGAN approaches can be integrated into cloud orchestration platforms such as OpenStack to enhance overload detection and VM placement. Its predictive capabilities can improve the OpenStack Nova scheduler for better VM allocation and optimize the OpenStack Watcher service for proactive workload balancing, reducing unnecessary migrations and enhancing system efficiency.

While this work focuses on overload detection within VM consolidation, future research will investigate VM selection and placement strategies, considering additional factors such as memory and disk storage. Further exploration of application-aware objectives, such as optimizing I/O operations, will improve data transfer efficiency and overall system performance. Additionally, integrating multi-criteria decision-making frameworks and online training methods can enable adaptive workload management in dynamic environments. Using real-world workload traces from Google and other cloud providers will provide deeper insights into performance variations and resource allocation challenges. Finally, reinforcement learning (RL) techniques will be explored to optimize VM migration and resource allocation strategies.

## Supplementary Information

Supplementary Information.

## Data Availability

The datasets used in the current research are available from the corresponding author upon individual request.
