# Supplementary material for: VTGAN based proactive VM consolidation in cloud data centers using value and trend approaches
Source: Sci Rep. 2025 Jun 20;15:20133. doi: 10.1038/s41598-025-04757-z (PMC12181298; doi:10.1038/s41598-025-04757-z)
Supplement: Supplementary file 1 — Supplementary Information. [file 41598_2025_4757_MOESM1_ESM.pdf]

Supplementary Material: Extended Background

Aya I. Maiyza, Hanan A. Hassan , Walaa M. Sheta, Karim Banawan, and Noha O. Korany

August 17, 2024

1 Extended Experimental configuration

The broad research challenge of RM and reallocation in the cloud has been subdivided and tackled by researchers from diverse perspectives [3, 38, 17]. Initially, efforts to reduce energy usage in data centers involved a strategy of periodically redistributing workloads, as discussed in studies such as [31, 40]. However, the authors did not employ an algorithm to identify optimal times to optimize virtual machine (VM) placement. Instead, they relied on a heuristic approach that was periodically triggered to adjust the VM placement.

An alternative solution involves using threshold-based heuristic strategies, as explored in studies such as [14, 44, 15, 1]. This approach monitors current behavior by initiating migrations from servers experiencing underload or overload when a specific threshold value is reached. Consequently, the VM consolidation approach emerged. The main goal of VM consolidation is to fulfill the resource requirements of VMs while consolidating their workloads into fewer physical machines. Researchers have approached the problem of VM consolidation from different perspectives, including VM placement [6, 12, 24, 38], overload and underload detection [4, 20, 42, 17], and VM selection for migrations [29, 30, 27].

In our study, we focus mainly on detecting overloaded servers. This decision-making challenge is complex and highly dependent on the type of workload and its variations over time.

Several approaches tackle this issue by determining if a host is overloaded or underloaded based on its current resource utilization compared to two static thresholds (hot and cold thresholds). For instance, Xiao et al. [41] propose a VM consolidation approach with fixed thresholds of 80% for overload and 40% for underload. The system then reallocates the migrated VMs to suitable destination hosts. Other researchers suggest using dynamic thresholds instead of static ones, arguing that static thresholds are insufficient for handling the dynamic nature of workloads in CDCs and may result in unnecessary migrations.

Hence, Beloglazov et al. [4] argued against maintaining a constant utilization threshold, citing the continuous fluctuations in workload utilization. They advocated for dynamic determination of the upper utilization threshold using methods like interquartile range (IQR) and median absolute deviation (MAD).

Overloaded servers were identified when their current load exceeded the upper utilization threshold. In the context of the dynamic cloud environment, they emphasized the efficiency of adaptive thresholds over fixed ones. The authors proposed techniques to predict CPU utilization, including local regression (LR) and robust local regression (LRR) algorithms. These methods were compared with dynamic thresholds to detect the overloaded server, revealing that LR led to lower EC and SLAVs than LRR.

In contrast to these methodologies, various studies advocate for proactive, dynamic VM consolidation algorithms. Depending only on the current host state might lead to unreliable decisions, potentially causing unnecessary migrations. Frequent migrations have the potential to negatively affect performance and introduce extra delays and downtime, thereby elevating the likelihood of SLA violations. As a result, predictive methods have been extensively investigated in the literature to anticipate future load fluctuations [5, 2] and adjust cloud resources based on future resource demands [33].

Recently, models for forecasting workload are commonly employed for predictive consolidation techniques. In general, researchers have examined the prediction of future resource usage in CDCs using historical data in previous studies [10, 21, 42]. Commonly, these studies utilize a workload forecasting model to guide VM live migration, considering either the predicted server state, the current state, or both. Farahnakian et al. [10] introduced a linear regression method to predict CPU utilization. Jheng et al. [21] applied the Grey model to forecast host CPU and RAM resource utilization. Despite its minimal data requirements and reliance on straightforward mathematical calculations, the experimental results revealed its limitations in ensuring reliable predictions for workloads with frequent fluctuations. Furthermore, Markov prediction has proven its effectiveness in forecasting statistical data characterized by frequent changes. Combining Markov prediction with the Grey forecasting model enhances prediction accuracy, particularly in systems managing highly variable time series. Consequently, in scenarios involving time series with frequent changes, the Grey–Markov prediction model outperforms the Grey prediction model [18]. Then, Hsieh et al. [20] enhanced the Grey-Markov model to predict the short-term future CPU utilization of hosts. The determination of overloaded and underloaded servers involves comparing their current and predicted CPU utilization with preset thresholds. Overload detection relies on a dynamic threshold derived from the median absolute deviation (MAD), whereas underload detection uses a static threshold. Sayadnavard et al. [35] presented a Discrete-Time Markov Chain (DTMC) approach for forecasting future resource utilization. Subsequently, they introduced a multi-objective VM placement strategy utilizing the e-dominance-based multi-objective artificial bee colony (e-MOABC) algorithm. This method efficiently manages energy usage, decreases resource wastage, and enhances system reliability to fulfil SLA and QoS demands.

Alternative studies leverage Machine Learning technology for resource usage prediction. In [23], an energy-efficient and SLA-aware consolidation approach is introduced, employing the Host States Naive Bayesian Prediction (HSNBP)

model in CDC. This model utilizes a naive Bayesian classifier to predict overload hosts. In [8], a method proposes predictive consolidation of virtual machines (PCVM) using an autoregressive integrated moving average (ARIMA) to optimize the deployment of VMs on a minimal number of servers. It aims to reduce unnecessary migrations, detect physical machine overload, and ensure SLAs through an ARIMA prediction model. The DVFS approach determines optimal frequencies for various physical devices. The experimental results show significant reductions in EC and improved QoS compared to baseline techniques. Linear Regression is commonly employed in various studies due to its simplicity for estimating future resource usage.

The methods proposed in [10, 9, 19, 36] used linear regression to predict CPU utilization for VM consolidation or placement. However, the linear regression technique only accounts for the linear relationships between the variables considered. The connection between resource demand data and time, however, seems to exhibit a more curved pattern. In the study described in [11], the authors substituted linear regression with K-Nearest-Neighbor (KNN) regression. They observed that this approach enhances energy saving and SLAV more effectively than linear regression.

In [22], a method known as self-directed workload forecasting (SDWF) was introduced. SDWF employs a Multi-layer Neural Network (MNN) to analyze historical utilization and forecast future workloads. It captures the trend of forecasting errors by calculating deviations in recent predictions, using this information to improve the accuracy of subsequent forecasts. This model employs an enhanced heuristic approach inspired by the black hole phenomena to train neurons. [34] introduced the Multi-resource Feedforward Neural Network (OM-FNN) to forecast future resource requirements in concurrent applications. OM-FNN utilizes the differential evolution algorithm to improve learning and prediction capabilities.

Similarly, Abdullah et al. [2] used Support Vector Regression (SVR) with the Radial Basis Function kernel and Sequential Minimal Optimization Algorithm (SMOA) for training to predict future usage of multi-attribute host resources. Thein et al. [39] introduced a framework that utilizes a combination of reinforcement learning (RL) mechanisms and fuzzy logic (FL) to address cloud resource allocation.

Some studies employ hybrid or ensemble learning approaches to improve the accuracy of predictions. For example, Ghobaei-Arani et al. [13] introduced a hybrid method that merges linear regression with reinforcement learning techniques to manage changes in the workload traces effectively. In a different approach, Liu et al. [26] used the ARIMA prediction model to detect host overload.

Liu et al. [25] integrated ARIMA with Long short-term memory (LSTM) models, demonstrating a 6% improvement over pure LSTM and a 66% improvement over pure ARIMA in prediction accuracy. Shuvo et al. [37] proposed the LSRU hybrid model, combining gated recurrent units (GRU) with LSTM, outperforming individual LSTM and GRU models in accuracy. Bi et al. [7] presented the BG-LSTM hybrid model, which integrates bidirectional and grid-

LSTM networks for enhanced accuracy.

The merging of ConvNets and LSTM is a popular hybrid approach to time series prediction [43]. Ouhame et al. [32] combined CNN with LSTM, extracting complex features of VM utilization components and modeling temporal characteristics of irregular patterns. The results demonstrated its superiority over other hybrid models.

The use of GANs in time-series cloud workload prediction is a recent development [16, 43]. Yazdanian and Sharifan [43] proposed the E2LG model, combining LSTM as a generator and CNN as a discriminator, effectively capturing long-term non-linear dependencies in high-frequency data. In [28], we used a modified version of GAN to forecast both the value and trend of the workload, transferring the problem from value regression to trend classification. Also, we studied the effect of using technical indicators (TIs), window input size, and multi-step prediction in the performance of our regression and classification models. In contrast to [43], our approach prioritizes predicting near-future utilization by promptly adapting to unexpected changes based on recent historical data [28].

References

[1] Vmware distributed power management: Concepts and usage, april 2013, tech. rep. `https://www.vmware.com/techpapers/2008/vmware-distributed-power-management-concepts-and-1080.html`. accessed(10/2018).

[2] Labeb Abdullah, Huixi Li, Shamsan Al-Jamali, Abdulrahman Al-Badwi, and Chang Ruan. Predicting multi-attribute host resource utilization using support vector regression technique. *IEEE Access*, 8:66048–66067, 2020. `https://doi.org/10.1109/ACCESS.2020.2984056`.

[3] Mirna Awad, Nadjia Kara, and Aris Leivadeas. Utilization prediction-based vm consolidation approach. *Journal of Parallel and Distributed Computing*, 170:24–38, 2022. `https://doi.org/10.1016/j.jpdc.2022.08.001`.

[4] Anton Beloglazov and Rajkumar Buyya. Optimal online deterministic algorithms and adaptive heuristics for energy and performance efficient dynamic consolidation of virtual machines in cloud data centers. *Concurrency and Computation: Practice and Experience*, 24(13):1397–1420, 2012. `https://doi.org/10.1002/cpe.1867`.

[5] Souhila Benmakrelouf, Nadjia Kara, Hanine Tout, Rafi Rabipour, and Claes Edstrom. Resource needs prediction in virtualized systems: Generic proactive and self-adaptive solution. *Journal of Network and Computer Applications*, 148:102443, 2019. `https://doi.org/10.1016/j.jnca.2019.102443`.

[6] G Bharanidharan and S Jayalakshmi. Predictive virtual machine placement for energy efficient scalable resource provisioning in modern data centers. In *2021 8th International Conference on Computing for Sustainable Global Development (INDIACom)*, pages 299–305. IEEE, 2021. https://ieeexplore.ieee.org/abstract/document/9441152.

[7] Jing Bi, Shuang Li, Haitao Yuan, and MengChu Zhou. Integrated deep learning method for workload and resource prediction in cloud systems. *Neurocomputing*, 424:35–48, 2021. https://doi.org/10.1016/j.neucom.2020.11.011.

[8] Maryam Chehelgerdi-Samani and Faramarz Safi-Esfahani. Pcvm. arima: predictive consolidation of virtual machines applying arima method. *The Journal of Supercomputing*, 77:2172–2206, 2021. https://doi.org/10.1007/s11227-020-03354-3.

[9] Fahimeh Farahnakian, Adnan Ashraf, Tapio Pahikkala, Pasi Liljeberg, Juha Plosila, Ivan Porres, and Hannu Tenhunen. Using ant colony system to consolidate vms for green cloud computing. *IEEE transactions on services computing*, 8(2):187–198, 2014. https://doi.org/10.1109/TSC.2014.2382555.

[10] Fahimeh Farahnakian, Pasi Liljeberg, and Juha Plosila. Lircup: Linear regression based cpu usage prediction algorithm for live migration of virtual machines in data centers. In *2013 39th Euromicro Conference on Software Engineering and Advanced Applications*, pages 357–364. IEEE, 2013. https://doi.org/10.1109/SEAA.2013.23.

[11] Fahimeh Farahnakian, Tapio Pahikkala, Pasi Liljeberg, and Juha Plosila. Energy aware consolidation algorithm based on k-nearest neighbor regression for cloud data centers. In *2013 IEEE/ACM 6th International Conference on Utility and Cloud Computing*, pages 256–259. IEEE, 2013. https://doi.org/10.1109/UCC.2013.51.

[12] Xiong Fu and Chen Zhou. Predicted affinity based virtual machine placement in cloud computing environments. *IEEE Transactions on Cloud Computing*, 8(1):246–255, 2017. https://doi.org/10.1109/TCC.2017.2737624.

[13] Mostafa Ghobaei-Arani, Sam Jabbehdari, and Mohammad Ali Pourmina. An autonomic resource provisioning approach for service-based cloud applications: A hybrid approach. *Future Generation Computer Systems*, 78:191–210, 2018. https://doi.org/10.1016/j.future.2017.02.022.

[14] Daniel Gmach, Jerry Rolia, Ludmila Cherkasova, Guillaume Belrose, Tom Turicchi, and Alfons Kemper. An integrated approach to resource pool management: Policies, efficiency and quality metrics. In *Dependable Systems and Networks With FTCS and DCC, 2008. DSN 2008. IEEE International Conference on*, pages 326–335. IEEE, 2008. https://doi.org/10.1109/DSN.2008.4630101.

[15] Daniel Gmach, Jerry Rolia, Ludmila Cherkasova, and Alfons Kemper. Resource pool management: Reactive versus proactive or let's be friends. *Computer Networks*, 53(17):2905–2922, 2009. https://doi.org/10.1016/j.comnet.2009.08.011.

[16] Ian J Goodfellow, Jean Pouget-Abadie, Mehdi Mirza, Bing Xu, David Warde-Farley, Sherjil Ozair, Aaron Courville, and Yoshua Bengio. Generative adversarial networks. *arXiv preprint arXiv:1406.2661*, 2014. https://doi.org/10.48550/arXiv.1406.2661.

[17] Hanan A Hassan, Aya I Maiyza, and Walaa M Sheta. Integrated resource management pipeline for dynamic resource-effective cloud data center. *Journal of Cloud Computing*, 9(1):1–20, 2020. https://doi.org/10.1186/s13677-020-00212-8.

[18] Yong He and YD Bao. Grey-markov forecasting model and its application. *System Engineering-Theory & Practice*, 9(4):59–63, 1992.

[19] Nguyen Trung Hieu, Mario Di Francesco, and Antti Ylä-Jääski. Virtual machine consolidation with usage prediction for energy-efficient cloud data centers. In *2015 IEEE 8th International Conference on Cloud Computing*, pages 750–757. IEEE, 2015. https://doi.org/10.1109/CLOUD.2015.104.

[20] Sun-Yuan Hsieh, Cheng-Sheng Liu, Rajkumar Buyya, and Albert Y Zomaya. Utilization-prediction-aware virtual machine consolidation approach for energy-efficient cloud data centers. *Journal of Parallel and Distributed Computing*, 139:99–109, 2020. https://doi.org/10.1016/j.jpdc.2019.12.014.

[21] Jhu-Jyun Jheng, Fan-Hsun Tseng, Han-Chieh Chao, and Li-Der Chou. A novel vm workload prediction using grey forecasting model in cloud data center. In *The International Conference on Information Networking 2014 (ICOIN2014)*, pages 40–45. IEEE, 2014. https://doi.org/10.1109/ICOIN.2014.6799662.

[22] Jitendra Kumar, Ashutosh Kumar Singh, and Rajkumar Buyya. Self directed learning based workload forecasting model for cloud resource management. *Information Sciences*, 543:345–366, 2021. https://doi.org/10.1016/j.ins.2020.07.012.

[23] Lianpeng Li, Jian Dong, Decheng Zuo, and JIaxi Liu. Sla-aware and energy-efficient vm consolidation in cloud data centers using host states naive bayesian prediction model. In *2018 IEEE Intl Conf on Parallel & Distributed Processing with Applications, Ubiquitous Computing & Communications, Big Data & Cloud Computing, Social Computing & Networking, Sustainable Computing & Communications (ISPA/IUCC/BDCloud/SocialCom/SustainCom)*, pages 80–87. IEEE, 2018. https://doi.org/10.1109/BDCloud.2018.00025.

[24] Zhihua Li, Xinrong Yu, Lei Yu, Shujie Guo, and Victor Chang. Energy-efficient and quality-aware vm consolidation method. *Future Generation Computer Systems*, 102:789–809, 2020. https://doi.org/10.1016/j.future.2019.08.004.

[25] Jing Liu, Xueyong Tan, and Yan Wang. Cssap: software aging prediction for cloud services based on arima-lstm hybrid model. In *2019 IEEE International Conference on Web Services (ICWS)*, pages 283–290. IEEE, 2019. https://doi.org/10.1109/ICWS.2019.00055.

[26] Yaqiu Liu, Xinyue Sun, Wei Wei, and Weipeng Jing. Enhancing energy-efficient and qos dynamic virtual machine consolidation method in cloud environment. *IEEE Access*, 6:31224–31235, 2018. https://doi.org/10.1109/ACCESS.2018.2835670.

[27] Aya I Maiyza, Hanan A Hassan, Walaa M Sheta, Nayra M Sadek, and Mohamed A Mokhtar. End-user's sla-aware consolidation in cloud data centers. In *2017 IEEE International Symposium on Signal Processing and Information Technology (ISSPIT)*, pages 196–204. IEEE, 2017. https://doi.org/10.1109/ISSPIT.2017.8388641.

[28] Aya I Maiyza, Noha O Korany, Karim Banawan, Hanan A Hassan, and Walaa M Sheta. Vtgan: hybrid generative adversarial networks for cloud workload prediction. *Journal of Cloud Computing*, 12(1):97, 2023. https://doi.org/10.1186/s13677-023-00473-z.

[29] Suhib Bani Melhem, Anjali Agarwal, Nishith Goel, and Marzia Zaman. Selection process approaches in live migration: A comparative study. In *Information and Communication Systems (ICICS), 2017 8th International Conference on*, pages 23–28. IEEE, 2017. https://doi.org/10.1109/IACS.2017.7921940.

[30] Seyedhamid Mashhadi Moghaddam, Sareh Fotuhi Piraghaj, Michael O'Sullivan, Cameron Walker, and Charles Unsworth. Energy-efficient and sla-aware virtual machine selection algorithm for dynamic resource allocation in cloud data centers. In *2018 IEEE/ACM 11th International Conference on Utility and Cloud Computing (UCC)*, pages 103–113. IEEE, 2018. https://doi.org/10.1109/UCC.2018.00019.

[31] Ripal Nathuji and Karsten Schwan. Virtualpower: coordinated power management in virtualized enterprise systems. *ACM SIGOPS operating systems review*, 41(6):265–278, 2007. https://doi.org/10.1145/1323293.1294287.

[32] Soukaina Ouhame, Youssef Hadi, and Arif Ullah. An efficient forecasting approach for resource utilization in cloud data center using cnn-lstm model. *Neural Computing and Applications*, pages 1–13, 2021. https://doi.org/10.1007/s00521-021-05770-9.

[33] EG Radhika and G Sudha Sadasivam. A review on prediction based autoscaling techniques for heterogeneous applications in cloud environment. *Materials Today: Proceedings*, 45:2793–2800, 2021. https://doi.org/10.1016/j.matpr.2020.11.789.

[34] Deepika Saxena and Ashutosh Kumar Singh. A proactive autoscaling and energy-efficient vm allocation framework using online multi-resource neural network for cloud data center. *Neurocomputing*, 426:248–264, 2021. https://doi.org/10.1016/j.neucom.2020.08.076.

[35] Monireh H Sayadnavard, Abolfazl Toroghi Haghighat, and Amir Masoud Rahmani. A multi-objective approach for energy-efficient and reliable dynamic vm consolidation in cloud data centers. *Engineering science and technology, an International Journal*, 26:100995, 2022. https://doi.org/10.1016/j.jestch.2021.04.014.

[36] Subhadra Bose Shaw, Jay Prakash Kumar, and Anil Kumar Singh. Energy-performance trade-off through restricted virtual machine consolidation in cloud data center. In *2017 International Conference on Intelligent Computing and Control (I2C2)*, pages 1–6. IEEE, 2017. https://doi.org/10.1109/I2C2.2017.8321783.

[37] Md Nahid Hasan Shuvo, Mirza Mohd Shahriar Maswood, and Abdullah G Alharbi. Lsru: A novel deep learning based hybrid method to predict the workload of virtual machines in cloud data center. In *2020 IEEE Region 10 Symposium (TENSYMP)*, pages 1604–1607. IEEE, 2020. https://doi.org/10.1109/TENSYMP50017.2020.9230799.

[38] Manoel C Silva Filho, Claudio C Monteiro, Pedro RM Inácio, and Mário M Freire. Approaches for optimizing virtual machine placement and migration in cloud environments: A survey. *Journal of Parallel and Distributed Computing*, 111:222–250, 2018. https://doi.org/10.1016/j.jpdc.2017.08.010.

[39] Thandar Thein, Myint Myat Myo, Sazia Parvin, and Amjad Gawanmeh. Reinforcement learning based methodology for energy-efficient resource allocation in cloud data centers. *Journal of King Saud University-Computer and Information Sciences*, 32(10):1127–1139, 2020. https://doi.org/10.1016/j.jksuci.2018.11.005.

[40] Akshat Verma, Puneet Ahuja, and Anindya Neogi. pmapper: power and migration cost aware application placement in virtualized systems. In *Proceedings of the 9th ACM/IFIP/USENIX International Conference on Middleware*, pages 243–264. Springer-Verlag New York, Inc., 2008. https://doi.org/10.1007/978-3-540-89856-6_13.

[41] Hui Xiao, Zhigang Hu, and Keqin Li. Multi-objective vm consolidation based on thresholds and ant colony system in cloud computing. *IEEE*

Access, 7:53441–53453, 2019. https://doi.org/10.1109/ACCESS.2019.2912722.

[42] Zhen Xiao, Weijia Song, and Qi Chen. Dynamic resource allocation using virtual machines for cloud computing environment. *IEEE transactions on parallel and distributed systems*, 24(6):1107–1117, 2012. https://doi.org/10.1109/TPDS.2012.283.

[43] Peyman Yazdanian and Saeed Sharifian. E2lg: a multiscale ensemble of lstm/gan deep learning architecture for multistep-ahead cloud workload prediction. *The Journal of Supercomputing*, pages 1–31, 2021. https://doi.org/10.1007/s11227-021-03723-6.

[44] Xiaoyun Zhu, Don Young, Brian J Watson, Zhikui Wang, Jerry Rolia, Sharad Singhal, Bret McKee, Chris Hyser, Daniel Gmach, Rob Gardner, et al. 1000 islands: Integrated capacity and workload management for the next generation data center. In *Autonomic Computing, 2008. ICAC'08. International Conference on*, pages 172–181. IEEE, 2008. https://doi.org/10.1109/ICAC.2008.32.
